# Oxidative Nanopatterning of Titanium Surface Influences mRNA and MicroRNA Expression in Human Alveolar Bone Osteoblastic Cells

**DOI:** 10.1155/2016/9169371

**Published:** 2016-04-21

**Authors:** Maidy Rehder Wimmers Ferreira, Roger Rodrigo Fernandes, Amanda Freire Assis, Janaína A. Dernowsek, Geraldo A. Passos, Fabio Variola, Karina Fittipaldi Bombonato-Prado

**Affiliations:** ^1^Cell Culture Laboratory, Department of Morphology, Physiology and Basic Pathology, School of Dentistry of Ribeirão Preto, University of São Paulo, 14040-904 Ribeirão Preto, SP, Brazil; ^2^Molecular Immunogenetics Group, Department of Genetics, Ribeirão Preto Medical School, University of São Paulo, 14049-900 Ribeirão Preto, SP, Brazil; ^3^Faculty of Engineering, Department of Mechanical Engineering, University of Ottawa, Ottawa, ON, Canada K1N 6N5

## Abstract

Titanium implants have been extensively used in orthopedic and dental applications. It is well known that micro- and nanoscale surface features of biomaterials affect cellular events that control implant-host tissue interactions. To improve our understanding of how multiscale surface features affect cell behavior, we used microarrays to evaluate the transcriptional profile of osteoblastic cells from human alveolar bone cultured on engineered titanium surfaces, exhibiting the following topographies: nanotexture (N), nano+submicrotexture (NS), and rough microtexture (MR), obtained by modulating experimental parameters (temperature and solution composition) of a simple yet efficient chemical treatment with a H_2_SO_4_/H_2_O_2_ solution. Biochemical assays showed that cell culture proliferation augmented after 10 days, and cell viability increased gradually over 14 days. Among the treated surfaces, we observed an increase of alkaline phosphatase activity as a function of the surface texture, with higher activity shown by cells adhering onto nanotextured surfaces. Nevertheless, the rough microtexture group showed higher amounts of calcium than nanotextured group. Microarray data showed differential expression of 716 mRNAs and 32 microRNAs with functions associated with osteogenesis. Results suggest that oxidative nanopatterning of titanium surfaces induces changes in the metabolism of osteoblastic cells and contribute to the explanation of the mechanisms that control cell responses to micro- and nanoengineered surfaces.

## 1. Introduction

Over the last three decades, orthopedics and oral and maxillofacial surgery have used titanium as the metallic material of choice because of its excellent biocompatibility, mainly associated with (1) elastic modulus similar to that of bone, (2) excellent corrosion resistance due to a superficial TiO_2_ layer, and (3) biological inertness* in vivo* [1]. These advantages have boosted the application of titanium, ranging from femoral stems to prosthetic devices to replace dental elements [2]. However, specific physiological aspects such as implantation site, blood supply, and quality and quantity of the surrounding bone tissue can interfere with the osseointegration process, ultimately determining the success rate of an implant [3]. In addition to these factors, the metal physicochemical properties (e.g., topography, roughness, chemical composition, and wettability) at various scales will also contribute to the determination of the outcome of the osseointegration process by affecting the cellular and extracellular events that occur during implant-host tissue interactions [4].

The abilities to promote the interactions with adjacent tissues and to elicit the biological response by guiding specific cellular processes along predetermined routes are fundamental characteristics that the next generation of biomaterials should possess [5]. It is now widely accepted that the rational design of surface topography at the micro- and nanoscale is a powerful tool to control and guide cellular response [6]. The topography of a surface can in fact influence cellular response from surrounding tissues by modifying cell adhesion and migration, proliferation, and collagen synthesis at the material-host tissue interface [7]. Similarly, surface chemistry is another key parameter that plays a fundamental role in peri-implant bone apposition [8].

Numerous techniques have been developed to engineer titanium surfaces in ways to promote bone cell growth and ultimately implant fixation. Several studies have shown how different types of titanium surface treatment affect these processes and highlighted how micro- and nanopatterned surfaces exert a differential influence on bone formation and cell behavior obtained from tissues adjacent to the implant surfaces [7]. In this context, cell cultures are a useful tool, because they allow investigation into how cells and matrices interact with the titanium surface [9]. Currently, the investigation of gene expression patterns is increasingly gaining interest, aiming at unveiling the functional roles of genes and enabling new approaches in cell therapies [10]. Tools such as microarrays can now be used to identify gene modulation in cells that are in contact with biomaterials, as reported by Bombonato-Prado et al. [11]. Microarrays can ultimately help to identify differentially regulated genes in osteoblasts exposed to different biomaterials used in bone regeneration/substitution procedures.

The present study relied on biochemical assays and gene expression to evaluate differences in the cellular response of human alveolar bone cells cultured on different titanium surfaces. Our results showed that nanoporous titanium surfaces generated by oxidative nanopatterning influence alveolar bone cells behavior and, distinctively from previous studies, there were investigated differences in the expression of mRNAs and microRNAs of such cells in contact with the distinct topographies.

## 2. Materials and Methods

### 2.1. Titanium Surfaces Preparation

Commercially pure grade 2 titanium (Ti) discs, with diameter of 13 mm and thickness of 2 mm, were polished with an Exakt 400 CS machine equipped with 320, 500, 800, 2500, and 4000 grits (Exakt Advanced Technologies, Germany) and successively polished with felt and abrasive particles of alumina paste (Al_2_O_3_) (0.05 mM). The titanium discs were sonicated in Extran® MA 02 (Merck Millipore, USA) 2% diluted in deionized water, followed by alcohol 70% and deionized water for 30 minutes each. Next, simple yet efficient chemical etching based on a mixture of sulfuric acid (H_2_SO_4_ at 36 N) and hydrogen peroxide (H_2_O_2(aq)_) was applied at varying relative concentrations of the acid and the peroxide, at different temperatures. This procedure afforded three different types of titanium surface, as described in a previous article [5]. Application of a fully programmable digital hot plate (EchoTherm*™* HS40, Torrey Pines Scientific, USA) with automatic feedback ensured temperature control. To obtain the nanotextured surface (N), the titanium discs were submitted to etching with 50 : 50 H_2_SO_4_ (36 N)/30% aqueous hydrogen peroxide (H_2_O_2(aq)_) at 25°C. To achieve the nano+submicrotextured surface (NS), the titanium discs were treated with 50 : 50 H_2_SO_4_ (36 N)/30% aqueous hydrogen peroxide (H_2_O_2(aq)_) at 50°C. Treatment of the titanium discs with 30% aqueous hydrogen peroxide (H_2_O_2(aq)_) solution alone at 50°C was used to obtain the rough microtextured (MR) surface. Untreated polished titanium discs served as control (C). Before experiments, treated and untreated (control) titanium discs were rinsed with deionized H_2_O, autoclaved, and air-dried. To confirm the presence of different surface topographies, the titanium discs were examined under a field emission scanning electron microscope (Zeiss LEO 440, Cambridge, England) operated at 15 kV.

### 2.2. Cell Culture

Human alveolar bone fragments were obtained from healthy adult donors with their informed consent, using the research protocols approved by the Committee of Ethics in Research of the School of Dentistry of the University of São Paulo (approval number 2011.1.1015.58.6). The osteoblastic cells were kept in culture flasks until subconfluence and then seeded over titanium discs in 24-well culture plates at a concentration of 2 × 10^4^ cells/well. The growth medium consisted of alpha-minimum essential medium (*α*-MEM; Invitrogen-Life Technologies, Grand Island, NY) supplemented with 10% fetal calf serum (Gibco-Life Technologies), gentamicin (Gibco) at 50 mg/mL, and fungizone (Gibco) at 0.3 mg/mL, added with ascorbic acid (Gibco-Life Technologies) at 5 mg/mL, *β*-glycerophosphate (Sigma-Aldrich, St. Louis, MO) at 7 mM, and dexamethasone (Sigma-Aldrich) at 10^−7 ^M. The cell cultures were kept at 37°C under humidified atmosphere containing 5% CO_2_ and 95% air. The culture medium was changed three times a week.

### 2.3. Cell Viability

Cell viability was assessed by MTT assay (3-[4,5-dimethylthiazol-2-yl]-2,5-diphenyltetrazolium bromide) 7, 10, and 14 days after the start of the culture. To this end, cells were incubated with 10% MTT (5 mg/mL) in culture medium at 37°C for 4 hours. The medium was then aspirated from the well, and 1 mL of isopropanol (0.04 N HCl in isopropanol) was added to each well. The plates were placed on a shaker for 5 minutes and 200 *μ*L of this solution was transferred to a 96-well plate. The optical density was read at 570 nm (*μ*Quant, BioTek Instruments, Winooski, VT, USA).

### 2.4. Alkaline Phosphatase Assay

Alkaline phosphatase activity was assayed as the release of thymolphthalein from thymolphthalein monophosphate; a commercial kit (Labtest Diagnóstica, MG, Brazil) was employed for this purpose. Briefly, 50 mL of thymolphthalein monophosphate was mixed with 0.5 mL of diethanolamine buffer (0.3 *μ*mol/mL, pH 10.1), and the resulting solution was kept at 37°C, for 2 min. After that, 50 mL of the lysate was added to each of the wells, which were kept at 37°C, for 10 min. Then, 2 mL of a solution containing Na_2_CO_3_ (0.09 *μ*mol/mL) and NaOH (0.25 *μ*mol/mL) was added for color development. After 30 min, the absorbance was measured at 590 nm. The alkaline phosphatase activity was calculated from a standard curve with thymolphthalein concentrations ranging from 0.012 to 0.4 *μ*mol of thymolphthalein/h/mL. Data are expressed as the alkaline phosphatase activity normalized by the total protein assay.

### 2.5. Mineralized Matrix Formation

Mineralized matrix formation was detected at day 21 by means of Alizarin Red S (Sigma-Aldrich) staining for areas rich in calcium. Attached cells were fixed in 10% formalin at 4°C for 2 h followed by one-hour immersion in alcohol for each increasing concentration (30%, 50%, 70%, and 100%). The next step was staining with 2% Alizarin Red S, pH 4.2, for 10 min. The calcium content was evaluated with a colorimetric method formerly described [12]. All biochemical data were compared by the Kruskal-Wallis and Mann-Whitney test. SPSS 17.0 statistical software was used, and differences with *p* ≤ 0.05 were considered statistically significant.

### 2.6. Total RNA Extraction

After 10 days, the total RNA of each culture was extracted with mirVana total RNA isolation kit® (Ambion, NY, USA), according to the manufacturer's instruction. UV spectrophotometry confirmed that the RNA preparations were free of proteins and phenol. RNA degradation was assessed by microfluidic electrophoresis with Agilent 6000 RNA Nano chips, conducted on an Agilent 2100 Bioanalyzer (Agilent Technologies, Santa Clara, CA, USA). Only RNA samples free of proteins and phenol and featuring an RNA Integrity Number (RIN) ≥ 9.0 were used.

### 2.7. Microarray Hybridization

#### 2.7.1. mRNA Expression Profiling with Agilent 4 × 44 K Human Oligoarrays

Changes in gene expression were evaluated with the Agilent one-color (Cy3 fluorochrome) microarray-based gene expression platform according to the manufacturer's instructions. Briefly, 500 ng of the individual total RNA was employed to synthesize double-stranded cDNA and cyanine 3 (Cy3) CTP labeled complementary amplified RNA (cRNA) by means of the Agilent Linear Amplification Kit (Agilent), according to the manufacturer's instructions. By using Agilent human 4 × 44 K oligonucleotide microarrays (Agilent), cyanine-labeled complementary RNA was hybridized to microarrays in SureHyb chambers (Agilent) in a rotator oven at 65°C, for 17 h. Each array contained 44,000 oligonucleotide probes covering the entire human functional genome. The arrays were washed according to the manufacturer's instructions and scanned with an Agilent DNA Microarray scanner.

#### 2.7.2. miRNA Expression Profiling with Agilent 8 × 15 K Mouse Oligoarrays

Briefly, the total RNA samples were labeled with Cy3 by using the Agilent miRNA Complete Labeling and Hybridization Kit (Agilent). To this end, 100 ng of total RNA was dephosphorylated by incubation with calf intestinal phosphatase at 37°C for 30 min, denatured in 100% DMSO at 100°C for 8 min, and labeled with perCp-Cy3 by using T4 ligase at 16°C for 2 h. Each labeled RNA sample was hybridized to an individual array on 8 × 15 K format Agilent human miRNA array slides. Each array contained probes for 720 human miRNAs. Hybridization was performed in SureHyb chambers (Agilent) at 55°C for 20 h, and the arrays were washed according to the manufacturer's instructions and scanned.

### 2.8. Microarray Data Analysis

The oligo-mRNA and oligo-miRNA array slides were scanned with a DNA microarray scanner (Agilent), and the hybridization signals were extracted by using the Agilent Feature Extraction software. The microarray numerical quantitative data were normalized to the 75th percentile and were analyzed through the GeneSpring GX bioinformatics platform (http://www.agilent.com/chem/genespring), according to the default instructions. For mRNA analysis, we used ANOVA statistical test (*p* ≤ 0.05) with a fold change ≥ 2.0 and, for microRNA analysis, we used ANOVA statistical analysis (*p* ≤ 0.01) with a fold change ≥ 1.5 [13]. A complete file that provides all of the mRNAs and miRNAs present in the arrays used in this study, as well as the experimental conditions, is available online at a public database (http://www.ebi.ac.uk/arrayexpress/), Array Express accession E-MTAB-3091 (for mRNA hybridization) and E-MTAB-3093 (for microRNA hybridization).

### 2.9. Oligonucleotide Primer Design and Quantitative Real-Time Polymerase Chain Reaction (qRT-PCR)

mRNA and microRNA oligoarray data were confirmed by qRT-PCR for five mRNAs (SMURF2, NOTCH1, PHOSPHO1, COL24A1, and FGF1) and six microRNAs (miR-31-3p, miR-134, miR-136-3p, miR-376c-3p, miR-424-5p, and miR-494). The mRNAs or microRNAs were elected on the basis of their expression pattern and biological function associated with the studied model system. The Primer3 web tool (http://biotools.umassmed.edu/bioapps/primer3_www.cgi) was used to select pairs of oligonucleotide primers with an optimal melting temperature of 60°C.  Table 1 lists the oligonucleotide primers used in qRT-PCRs primers for mRNAs and Table 2 lists the accession number and mature sequences for microRNAs. All the qRT-PCRs experiments were conducted in triplicate. One-way ANOVA statistical test was performed with the statistical software GraphPad Prism 5.0 (http://www.graphpad.com/prism/Prism.htm).

## 3. Results

### 3.1. Scanning Electron Microscopy (SEM)

**Figure 1:**
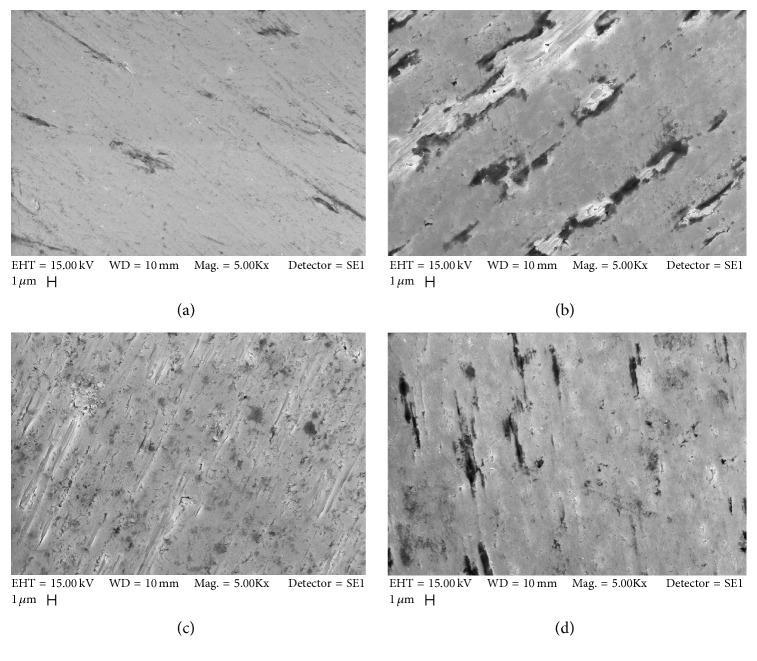
Scanning electron microscopy of different titanium surfaces: (a) control surface, (b) nanotextured surface, (c) nano+submicrotextured surface, and (d) rough microtextured surface.

SEM helped to characterize the surface morphology of the titanium discs. The polished control titanium disc displayed smooth surface. The treated titanium discs presented topographic surfaces bearing nanocavities with distinct distribution along the disc surface. The nano+submicrotextured surface group contained the greatest number of nanocavities. Except for surface roughness, the rough microtextured surface group and the nanotextured surface group had quite similar surface images (Figure 1).

### 3.2. Cell Viability

**Figure 2:**
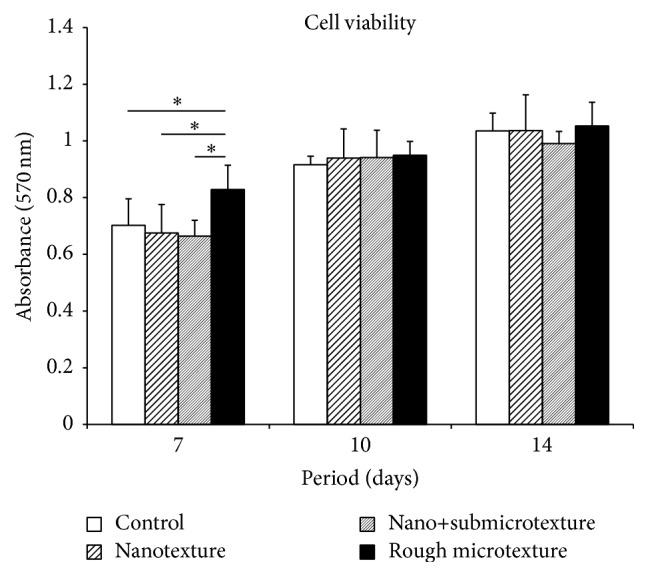
Cell viability of human alveolar osteoblastic cells at days 7, 10, and 14 of the start of the culture on polished titanium discs and on titanium surfaces etched by oxidative nanopatterning. Mann-Whitney test for ^*∗*^ *p* ≤ 0.05.

Cell viability was similar for all the groups at the various time points. The exception was the rough microtextured surface group, which exhibited significantly higher viability at day 7 after the start of the culture (*p* ≤ 0.05) as compared with the other groups (Figure 2).

### 3.3. Alkaline Phosphatase Activity

**Figure 3:**
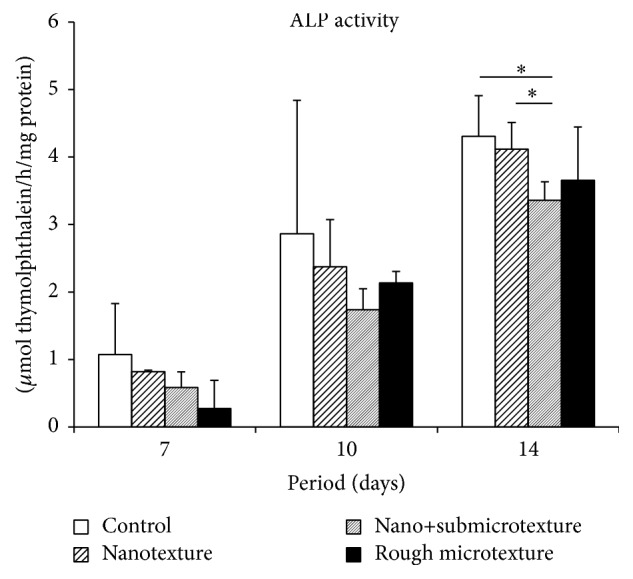
Alkaline phosphatase (ALP) activity of human alveolar osteoblastic cells after 7, 10, and 14 days of the start of the culture on polished titanium discs and on titanium surfaces etched by oxidative nanopatterning. Mann-Whitney test for ^*∗*^ *p* ≤ 0.05.

In all the experimental groups, the activity of the enzyme alkaline phosphatase, related to the mineralization process, was higher at day 14 after the start of the culture (Figure 3). Among the treated groups, the nanotextured surface group showed the highest alkaline phosphatase production, which was statistically significant as compared with the nano+submicrotextured group at day 14 after the start of the culture (*p* ≤ 0.05).

### 3.4. Mineralized Nodules

**Figure 4:**
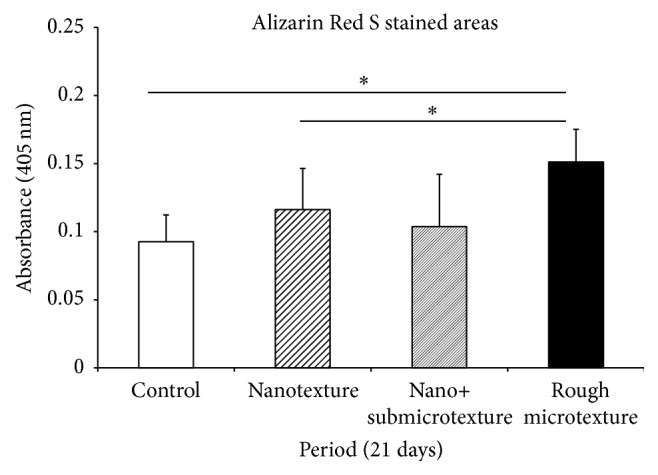
Quantitative analysis of Alizarin Red S stained areas of calcified nodules 21 days after the start of the culture on polished titanium discs and on titanium surfaces etched by oxidative nanopatterning. Mann-Whitney test for ^*∗*^ *p* ≤ 0.05.

Based on Alizarin Red S quantification, the treated titanium surfaces contained different amounts of calcium deposits. The rough microtextured surface group had the greatest amount of calcium as compared with the control group and the nanotextured surface group (*p* ≤ 0.05) (Figure 4).

### 3.5. Analysis of Differentially Expressed mRNAs

**Figure 5:**
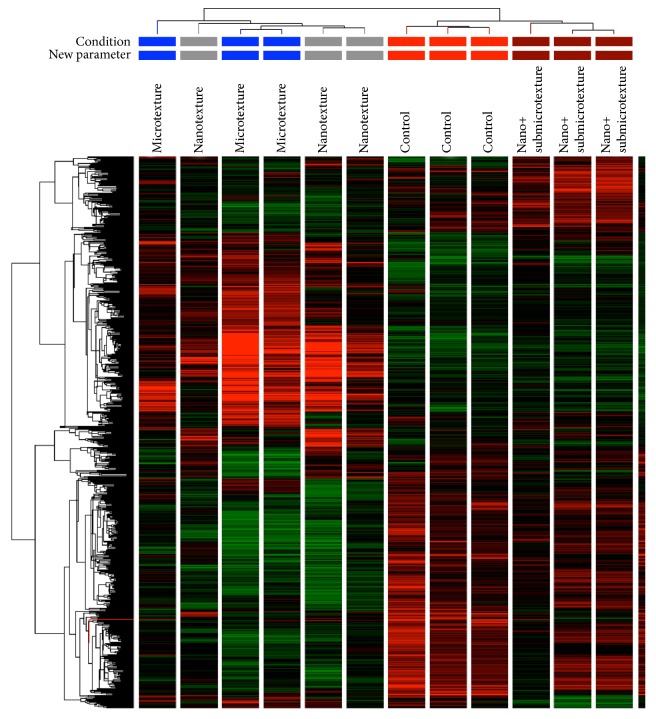
Hierarchical clustering of 716 differentially expressed genes of human alveolar osteoblastic cells on polished and etched titanium surfaces (nanotextured, nano+submicrotextured, and rough microtextured) 10 days after the start of the culture. Red: upregulation; green: downregulation; black: unmodulated.

A total of 716 genes showed fold change ≥ 2.0 and *p* ≤ 0.05 (Figure 5).  Table 3 displays differentially expressed genes associated with osteogenesis, cell adhesion, apoptosis, cell growth, and cell differentiation.  Table 4 depicts the differences in the expression of these genes as a function of the titanium surface topography.

### 3.6. Analysis of Differentially Expressed MicroRNAs

**Figure 6:**
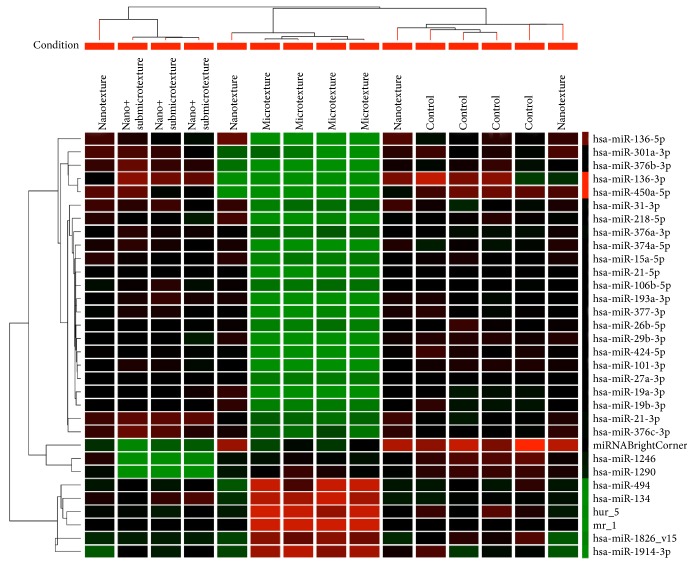
Hierarchical clustering of 32 differentially expressed microRNAs of human alveolar osteoblastic cells on polished and etched titanium surfaces (nanotextured, nano+submicrotextured, and rough microtextured) 10 days after the start of the culture. Red: upregulation; green: downregulation; black: unmodulated.

According to the results, 32 miRNAs showed fold change ≥ 1.5 and *p* ≤ 0.01 (Figure 6).  Table 5 presents differentially expressed miRNAs associated with osteogenesis, apoptosis, and cell growth.  Table 6 summarizes the different expression of these miRNAs in the studied titanium surfaces.

### 3.7. mRNA Data Confirmation by Real-Time Quantitative PCR

**Figure 7:**
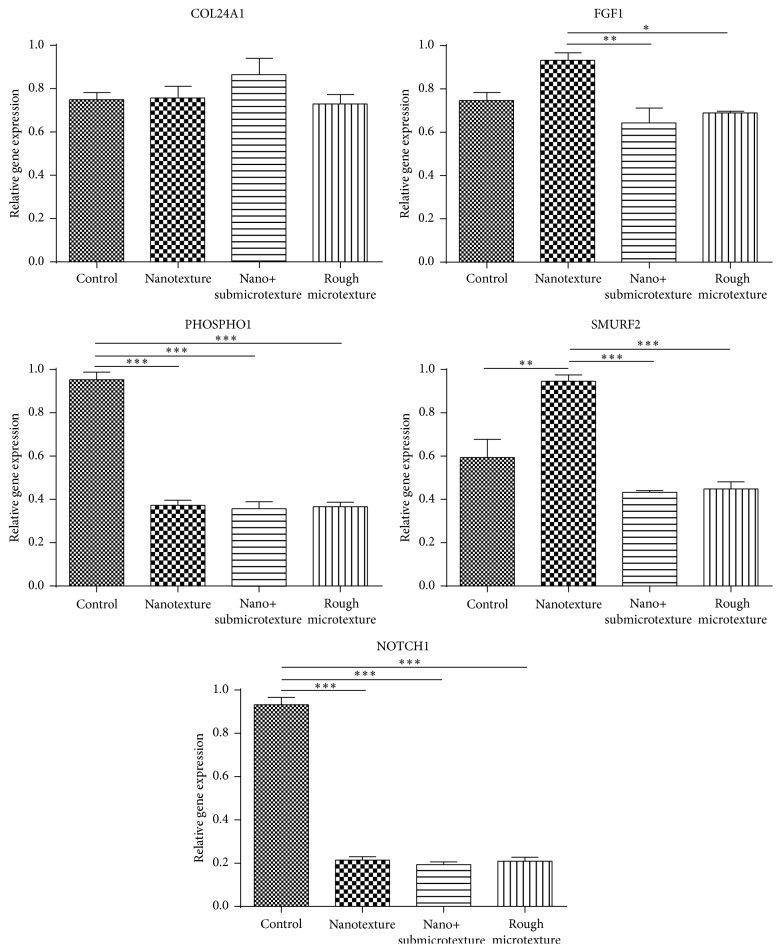
Quantitative expression of mRNAs of human alveolar osteoblastic cells on polished and etched titanium surfaces (nanotextured, nano+submicrotextured, and rough microtextured). Statistical analysis by Tukey Multiple Comparison Test; ^*∗*^ *p* < 0.05, ^*∗∗*^ *p* < 0.01, and ^*∗∗∗*^ *p* < 0.001.

qRT-PCR confirmed five differentially expressed mRNAs in the experimental groups; these mRNAs had been previously detected by oligomicroarray analysis (Table 5). The confirmed mRNAs are associated with apoptosis (NOTCH1), bone tissue and bone tissue mineralization (SMURF2, NOTCH1, and PHOSPHO1), cell adhesion (COL24A1 and FGF1), and cell proliferation (FGF1) (http://geneontology.org/, accessed 20/02/2014) (Figure 7).

### 3.8. MicroRNA Data Confirmation by Real-Time Quantitative PCR

**Figure 8:**
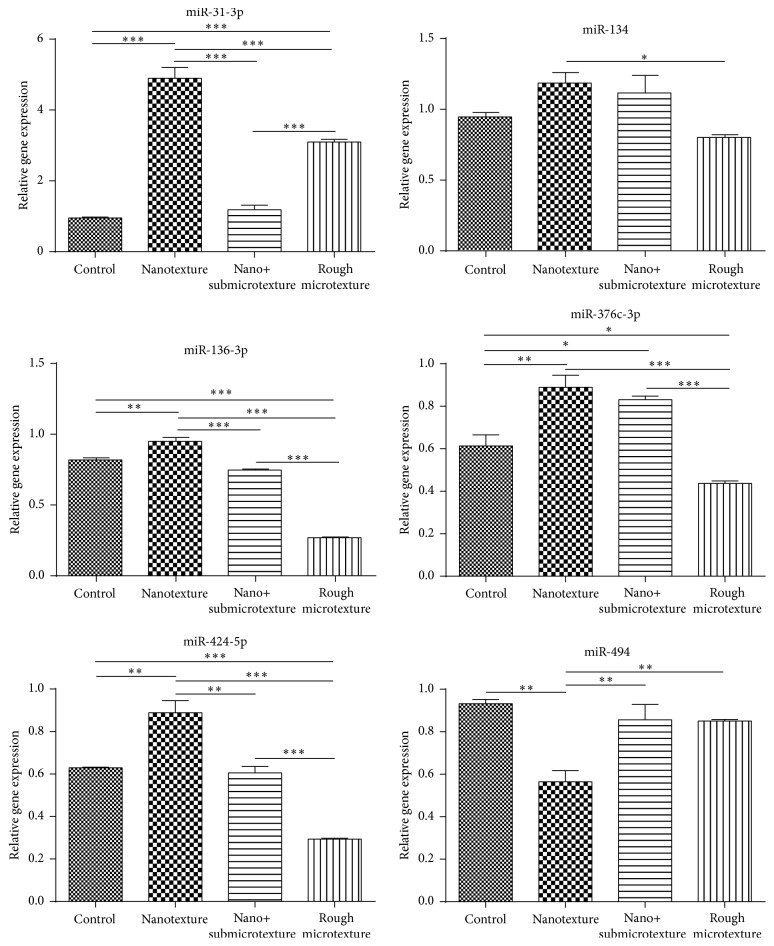
Quantitative expression of miRNAs of human alveolar osteoblastic cells on polished and etched titanium surfaces (nanotextured, nano+submicrotextured, and rough microtextured). Statistical analysis by Tukey Multiple Comparison Test; ^*∗*^ *p* < 0.05, ^*∗∗*^ *p* < 0.01, and ^*∗∗∗*^ *p* < 0.001.

Real-time quantitative PCR allowed analysis of six miRNAs that were differentially expressed in the experimental groups: miR-31-3p, miR-134, miR-136-3p, miR-376C-3p, miR-494, and miR-424-5p (Table 6). These miRNAs are associated with several functions like apoptosis (miR-134 and miR-494), bone mineralization (miR-31-3p, miR-136-3p, miR-376C-3p, and miR-424-5p), and cell growth and proliferation (miR-134) (http://geneontology.org/, accessed 02/20/2014) (Figure 8).

## 4. Discussion

Several studies aimed at analyzing and comparing the response of different cell types in contact with titanium surfaces modified by numerous methods [14]. In the present study, it was observed that oxidative nanopatterning of titanium engenders micro- and nanotextured surfaces that influence the metabolism of human alveolar bone cells. It has been shown that [15] nanotextured titanium surfaces generated by this chemical method promote osteoblast proliferation, making this a promising technique for the regulation of cellular activities in biological environments. A previous investigation revealed that chemical oxidation with H_2_SO_4_^conc^/H_2_O_2_^aq^ solution is an efficient tool to achieve various physical and chemical configurations on titanium surface, demonstrating that, by varying etching parameters such as solution composition, temperature, and exposure time, it is possible to modify the topography, oxide thickness, and wettability of commercially pure titanium [5]. In the present study, by using these parameters, we showed that oxidative nanopatterning promotes similar responses for cell viability for all groups at all time points tested, except for the rough microtexture, which showed significantly higher viability compared to the other groups after 7 days. Besides viability, biochemical assays like alkaline phosphatase activity are important to associate biomaterials topography with cell differentiation and osseointegration [8]. The ALP is among the first functional genes expressed in the calcification process, so it is possible that one of their roles in the mineralization process occurs at an early stage [16]. Nevertheless, the increased production of alkaline phosphatase was observed just after 14 days of culture in all experimental groups. Among the etched surfaces, cells cultured on nanotextured titanium discs showed the highest ALP activity, which was statistically significant compared to the NS group after 14 days. By exploiting microarray methodology, we found 716 differentially expressed genes of alveolar bone osteoblastic cells in contact with different titanium surfaces, most of them associated with the process of osteogenesis (e.g., mineralization, adhesion, apoptosis, proliferation, and differentiation). Among them, it was observed that NOTCH1 gene increased its expression in nano+submicrotexture surfaces when compared to the other experimental conditions. NOTCH is a key target in the osteoblastic cells and in osteoclastogenesis, as well as skeletal development and bone remodeling [17]. NOTCH is an additional pathway that is triggered early in the response to modified titanium surfaces, as it would be involved in the process of osteogenesis and* in vivo* bone formation and healing [18]. These findings are in agreement with our microarray results, despite the fact that qRT-PCR validation method showed similar responses only in the increased expression between C/MR and C/N groups. Another differentially expressed gene was PHOSPHO1, which showed higher expression in cells adhering onto rough microtexture. This gene codifies a protein involved in the initial deposition of hydroxyapatite crystals in early events of matrix mineralization [19]. Interestingly, the microarray data were in good agreement with our biochemical results, where the cultures in contact with rough microtexture showed higher calcium deposition, despite not being similar to qRT-PCR validation method. SMURF2 interacts with SMADS and induces ubiquitin-mediated degradation, preventing the signaling of TGF-*β* (transforming growth factor) and BMP (bone morphogenetic protein). Both the TGF-*β* and BMP are multifunctional proteins important for regulation of proliferation, differentiation, migration, and apoptosis [20]. Microarray data showed a higher expression of SMURF2 in cells adhering onto nano+submicrotexture surfaces when comparing to the other etched surfaces, whereas qRT-PCR revealed a higher expression of cells seeded on nanotextured titanium. These results suggest that the consequently lower expression of SMURF2 in cells seeded on MR surfaces might influence positively the osteoblast differentiation and Runx2 stability, promoting cell-biomaterial interaction, as seen after 7 and 21 days in MTT and Alizarin Red S staining assays, respectively. Another gene important to cell adhesion and cell proliferation is FGF1, which plays a crucial role in the proliferation and differentiation of osteoblasts [21]. FGF1 expression in microarray was increased in cells cultivated on control and NS surfaces, whereas qRT-PCR showed higher expression in cells seeded on nanotextured discs. Our biochemical assays on proliferation and viability did not reveal differences on cell adhesion and proliferation. COL24A1 gene proved to have some control on osteoblast differentiation and mineralization, through interaction with integrin *β*3 and the transforming growth factor beta (TGF-*β*)/SMADS signaling pathway [22]. It was found that COL24A1 gene is activated at the same time as the gene encoding osteocalcin, and its expression increases gradually as osteoblasts begin to deposit mineralized matrix [23]. Our microarray analysis showed increased expression in cells seeded on nanotexture titanium discs when compared to the other etched surfaces, which is in agreement with other investigations [24].

It has been observed that during bone formation several miRNAs participate both in early and in late stage of osteoblast differentiation regulating pathways* in vivo* [25]. The present study identified 32 miRNAs differentially expressed among the experimental groups. In particular, these miRNAs are involved in mineralization, apoptosis, and regulation of cell growth and proliferation. We showed that miR-136-3p exhibits a lower expression in cells seeded on rough microtexture surfaces when compared to N and MR surfaces in microarray and qRT-PCR data. The literature shows that miR-136 promotes downregulation in osteoblast differentiation [26], suggesting that in the present work this microRNA benefited the deposition of extracellular matrix in MR group as seen in our biochemical results. On the other hand, the higher expression of miR-134 in MR group revealed by microarray data may have resulted in diminished cell adhesion, as some microRNAs promote regulation of integrins, which are transmembrane cell adhesion receptors and have great importance for the unity of the cell to extracellular matrix, as well as participating in interactions between cells [26]. Poitz et al. [27] demonstrated that miR-134 promoted *β*1 integrin negative regulation, resulting in reduction of adhesion to fibronectin of mesenchymal stem cells (MSCs). Despite that, our MTT assay does not show any decrease in the viability of cells seeded on MR surface. Microarray and qRT-PCR methods also showed an increased expression of miR-494 in rough microtexture when compared to nanotexture. This microRNA mediates apoptosis and necrosis in different cell types [28], but this effect was not seen in the present work, with all cells seeded on etched surfaces showing similar or higher viability than control cells. We observed involvement of miR-31 in the regulation of transcription factor osterix in human bone marrow cells (MSCs) differentiated into osteoblasts [29]. Osterix is a key regulator of bone cell differentiation and plays an essential role in bone homeostasis [30]. Deng et al. [31] also observed that overexpression of miR-31 significantly reduced the expression of osteogenic transcription factors like OPN, BSP, OCN, and OSX, but not Runx2. Our data from microarray analysis as well as from qRT-PCR revealed that miR-31-3p had an increase in its expression in cells seeded on nanotexturized surface and a decrease on MR titanium discs. In the same way, cells on rough microtextured surface showed lower expression of miR-424-5p when compared to other etched surfaces whereas nanotextured surface revealed higher expression, confirmed by microarray and qRT-PCR. miR-424 also has regulatory roles in the differentiation of human bone marrow cells into osteoblasts [32] and these results may have contributed to the enhanced cell differentiation seen in the first group by means of ALP activity after 14 days. Other miRNAs, which influence bone metabolism, were differentially expressed in our study, such as miR-19b, miR-21, miR-218, and miR-29b.

The qRT-PCR method helps to validate the microarray data. In this study, we validated microarray methodology by conducting qRT-PCR of five genes and six microRNAs elected on the basis of their expression pattern and of their association with osteogenesis. However, qRT-PCR did not confirm all the results obtained by microarray analysis, which is shown also by other studies [33, 34]. According to the literature, differences between data obtained by the two methods may occur because microarray hybridization protocol might avoid the detection of subtle differences in gene expression, which could be detected by qRT-PCR technique [35]. Another cited reason is that these differences could occur as a consequence of increased separation between the locations of the PCR primers and the microarray probes [36]. Besides, microarray and qRT-PCR protocols perform normalization by different software (i.e., global gene expression and endogenous control, resp.) [37].

The results shown in this investigation reveal that several other mRNAs and miRNAs can be modulated as a consequence of surface modification, and more studies should be addressed to elucidate their role in osteoblast metabolism. In conclusion, it has been shown that oxidative nanopatterning of titanium surface influences alveolar bone osteoblastic cell metabolism and modulates the expression of genes encoding proteins that are important for osteogenesis.

## Figures and Tables

**Table 1:** Primers used in qRT-PCR reactions and their respective sense and antisense sequences.

mRNA symbol	Primer	Sequences	*T*_*m*_
SMURF2	Forward	5′-CATGTCTAACCCCGGAGGC-3′	60°C
	Reverse	5′-TCCATCAACCACCACCTTAGC-3′	
NOTCH1	Forward	5′-TACAAGTGCAACTGCCTGCT-3′	60°C
	Reverse	5′-ATAGTCCTCGGATTGCCTGC-3′	
PHOSPHO1	Forward	5′-ATACCTCAGCTAGCCCCCTT-3′	60°C
	Reverse	5′-TGTAGGGACTCTGTTGGCCT-3′	
COL24A1	Forward	5′-CCCCACGGCAAAAACGAAAT-3′	60°C
	Reverse	5′-GCCTCCAAGGCCTAGTTGAT-3′	
FGF1	Reverse	5′-ACGGGCTTTTATACGGCTCA-3′	60°C
	Forward	5′-ATGGTTCTCCTCCAGCCTTTC-3′	
GAPDH	Forward	5′-GGGTGTGAACCACGAGAAAT-3′	60°C
	Reverse	5′-CCTTCCACAATGCCAAAGTT-3′	

**Table 2:** Accession numbers and mature sequences of the microRNAs used in qRT-PCR reactions.

MicroRNA	ID-miRBASE	Sequences
hsa-miR-31-3p	MIMAT0004504	UGCUAUGCCAACAUAUUGCCAU
hsa-miR-134	MI0000474	CAGGGUGUGUGACUGGUUGACCAGAGGGGCAUGCACUGUGUUCACCCUGUGGGCCACCUAGUCACCAACCCUC
hsa-miR-136-3p	MIMAT0004606	CAUCAUCGUCUCAAAUGAGUCU
hsa-miR-376c-3p	MIMAT0000720	AACAUAGAGGAAAUUCCACGU
hsa-miR-424-5p	MIMAT0001341	CAGCAGCAAUUCAUGUUUUGAA
hsa-miR-494	MI0003134	GAUACUCGAAGGAGAGGUUGUCCGUGUUGUCUUCUCUUUAUUUAUGAUGAAACAUACACGGGAAACCUCUUUUUUAGUAUC

**Table 3:** Genes differentially expressed among the following groups: control, nanotexture, nano+submicrotexture, and rough microtexture after 10 days of culture, with associated functions according to the Gene Ontology database.

Osteogenesis	Cell adhesion	Apoptosis	Cell growth and proliferation	Cell differentiation
ATP6VOA4	AMICA1	AIPL1	E2F5	C10orf27
CDX1	SORBS1	LUC7L3	KIAA1109	SOX21
CYP27B1	NRXN1	ERBB3	LGI1	CYP24A1
SMURF2	PCDHA11	NOTCH1	CYP27B1	NANOG
NOTCH1	NRXN1	PSMD3	GRIN2A	HSF4
PHOSPHO1	FGF1	BTK	GPAM	LAMC3
	ERBB3	PTPRC	KDR	
	PCDHB10	ABCB9	MMP7	
	EPB41L5	KDR	PDE3A	
	UTRN	MAPT	CENPF	
	NOTCH1	GPAM	FGF1	
	NCKAP1L	RNF7	MECOM	
	COL24A1	CD28	PTPRK	

**Table 4:** Relative expression levels of mRNAs differentially expressed among control (C), nanotexture (N), nano+submicrotexture (NS), and rough microtexture (MR) groups after 10 days of culture. FC: fold change.

Gene	C versus MR	C versus S	C versus N	MR versus NS	MR versus N	NS versus N
	FC	FC	FC	FC	FC	FC
ABCB9	2,423	1,045	1,106	−2,319	−2,191	1,059
AIPL1	1,055	−2,369	1,164	−2,500	1,103	2,758
AMICA1	2,308	1,693	2,524	−1,364	1,094	1,491
ATP6V0A4	−5,137	−1,532	−3,833	3,353	1,340	−2,502
BTK	−4,130	−1,516	−6,530	2,725	−1,581	−4,308
C10orf27	−2,049	−1,277	−1,827	1,605	1,122	−1,431
CD28	−3,104	−1,478	−2,068	2,101	1,501	−1,400
CDX1	−2,178	−1,136	−1,572	1,918	1,386	−1,384
CENPF	2,296	1,639	1,574	−1,401	−1,459	−1,041
COL24A1	2,308	1,076	−1,584	−2,144	−3,655	−1,705
CYP27B1	2,325	−1,042	4,278	−2,422	1,840	4,457
E2F5	2,572	1,012	3,240	−2,542	1,260	3,203
EPB41L5	2,725	1,796	2,440	−1,518	−1,117	1,359
ERBB3	−2,036	1,391	−1,934	2,832	1,052	−2,691
FGF1	1,929	1,309	2,792	−1,473	1,447	2,132
GPAM	3,438	−1,467	2,420	−5,045	−1,421	3,551
GRIN2A	2,065	−1,066	2,005	−2,202	−1,030	2,138
HSF4	−2,358	−2,213	−1,331	1,066	1,772	1,663
KDR	1,563	−1,134	2,118	−1,772	1,355	2,401
KIAA1109	1,948	1,340	2,146	−1,453	1,102	1,602
LAMC3	2,138	2,921	2,086	1,366	−1,025	−1,400
LGI1	3,533	1,452	4,124	−2,433	1,167	2,839
LUC7L3	2,064	1,673	1,507	−1,234	−1,370	−1,110
MAPT	6,567	1,653	4,711	−3,973	−1,394	2,849
MECOM	−2,952	−3,421	−8,666	−1,159	−2,936	−2,533
MMP7	2,731	1,120	2,208	−2,438	−1,237	1,972
NANOG	3,365	2,137	3,791	−1,575	1,127	1,774
NCKAP1L	−1,722	−2,257	−1,736	−1,311	−1,008	1,300
NOTCH1	1,306	−1,812	1,416	−2,367	1,084	2,565
NRXN1	2,462	1,377	−1,203	−1,788	−2,963	−1,657
PCDHA11	2,074	−1,266	2,259	−2,626	1,089	2,859
PCDHB10	3,328	−1,025	3,581	−3,411	1,076	3,669
PDE3A	−3,260	1,121	−3,908	3,656	−1,199	−4,382
PHOSPHO1	−2,024	−1,214	−1,757	1,667	1,152	−1,448
PSMD3	−2,075	−2,131	−1,353	−1,027	1,533	1,574
PTPRC	1,453	−1,392	1,475	−2,022	1,016	2,054
PTPRK	−3,909	−1,952	1,034	2,002	4,040	2,018
RNF7	2,803	1,199	1,387	−2,339	−2,021	1,157
SMURF2	1,193	−2,344	−1,142	−2,797	−1,362	2,053
SORBS1	−3,880	−3,008	−3,342	1,290	1,161	−1,111
SOX21	2,006	1,583	2,829	−1,267	1,411	1,787
UTRN	−1,602	−1,884	−2,062	−1,176	−1,287	−1,094

**Table 5:** MicroRNAs differentially expressed among control, nanotexture, nano+submicrotexture, and rough microtexture groups after 10 days of culture, with associated functions according to Gene Ontology database.

Osteogenesis	Apoptosis	Cell growth and proliferation
hsa-miR-424-5p	hsa-miR-494	hsa-miR-134
hsa-miR-136-3p	hsa-miR-134	
hsa-miR-136-5p		
hsa-miR-31-3p		
hsa-miR-376c-3p		
hsa-miR-19b-3p		
hsa-miR-21-3p		
hsa-miR-21-5p		
hsa-miR-218-5p		
hsa-miR-29b-3p		

**Table 6:** Relative expression levels of microRNAs differentially expressed among control (C), nanotexture (N), nano+submicrotexture (NS), and rough microtexture (MR) groups after 10 days of culture. FC: fold change.

miRNA	C versus MR	C versus NS	C versus N	MR versus NS	MR versus N	NS versus N
	FC	FC	FC	FC	FC	FC
hsa-miR-101-3p	2,635	1,033	1,036	−2,551	−2,542	1,003
hsa-miR-106b-5p	1,511	−1,018	1,000	−1,539	−1,510	1,019
hsa-miR-1246	1,173	1,963	1,133	1,674	−1,036	−1,733
hsa-miR-1290	1,057	2,161	1,113	2,045	1,053	−1,941
hsa-miR-134	−1,478	−1,119	1,025	1,321	1,515	1,147
hsa-miR-136-3p	182,144	−1,044	4,031	−190,073	−45,188	4,206
hsa-miR-136-5p	67,552	1,001	−1,132	−67,496	−76,442	−1,133
hsa-miR-15a-5p	1,633	1,009	−1,027	−1,619	−1,678	−1,036
hsa-miR-1826_v15.0	−1,174	1,173	1,299	1,377	1,525	1,107
hsa-miR-1914-3p	−1,401	−1,001	1,186	1,399	1,662	1,188
hsa-miR-193a-3p	2,493	−1,059	−1,021	−2,640	−2,546	1,037
hsa-miR-19a-3p	2,033	−1,060	−1,069	−2,154	−2,173	−1,009
hsa-miR-19b-3p	1,544	−1,035	−1,053	−1,598	−1,627	−1,018
hsa-miR-21-3p	1,402	−1,205	−1,097	−1,688	−1,538	1,098
hsa-miR-21-5p	1,782	−1,000	−1,000	−1,782	−1,782	−1,000
hsa-miR-218-5p	1,931	1,054	−1,005	−1,832	−1,940	−1,059
hsa-miR-26b-5p	1,635	1,036	1,024	−1,579	−1,598	−1,012
hsa-miR-27a-3p	1,588	−1,000	−1,000	−1,588	−1,588	−1,000
hsa-miR-29b-3p	2,128	1,083	1,014	−1,964	−2,098	−1,068
hsa-miR-301a-3p	1,685	−1,041	1,034	−1,754	−1,630	1,076
hsa-miR-31-3p	1,464	−1,092	−1,120	−1,599	−1,640	−1,026
hsa-miR-374a-5p	1,868	−1,083	−1,093	−2,023	−2,041	−1,009
hsa-miR-376a-3p	1,411	−1,081	−1,046	−1,526	−1,477	1,033
hsa-miR-376b-3p	2,361	−1,105	1,085	−2,608	−2,176	1,199
hsa-miR-376c-3p	1,424	−1,141	−1,077	−1,625	−1,533	1,060
hsa-miR-377-3p	1,754	−1,019	1,005	−1,788	−1,745	1,025
hsa-miR-424-5p	2,125	1,051	1,043	−2,021	−2,038	−1,008
hsa-miR-450a-5p	202,710	1,121	3,964	−180,812	−51,138	3,536
hsa-miR-494	−1,416	1,006	1,120	1,424	1,585	1,113
hur_5	−1,357	1,120	1,132	1,519	1,537	1,011
miRNABrightCorner30	1,657	2,170	1,197	1,309	−1,385	−1,813
mr_1	−1,519	−1,000	−1,000	1,519	1,519	−1,000
